# Entropy and Human Aging

**DOI:** 10.1111/acel.70292

**Published:** 2025-11-13

**Authors:** Steven R. Cummings, Namki Hong, Alan A. Cohen

**Affiliations:** ^1^ San Francisco Coordinating Center California Pacific Medical Center Research Institute San Francisco California USA; ^2^ Department of Epidemiology and Biostatistics University of California San Francisco California USA; ^3^ Division of Endocrinology, Department of Internal Medicine, Severance Hospital Yonsei University College of Medicine Seoul South Korea; ^4^ Department of Environmental Health Sciences, Butler Columbia Aging Center, Mailman School of Public Health Columbia University New York USA

**Keywords:** aging, electrocardiography, entropy, rejuvenation

## Abstract

Entropy, a measure of disorder and randomness, is an essential feature of thermodynamics, chemistry, and information theory, but there has been little study of entropy in human aging. Entropy arises from random molecular interactions or other forms of damage and will manifest at all levels of human biology. It should also progress in concert across many systems and increase the risk of numerous aging‐related conditions. Illustrating these principles, research by Hong in this issue of *Aging Cell* applies Mahalanobis distance to quantify entropy in electrocardiograms showing that entropy in one system predicts aging‐related outcomes—fracture and mortality—beyond the heart. Important issues for research on entropy and human aging include the best methods for quantifying entropy and whether the development of entropy can be slowed or reversed in humans.

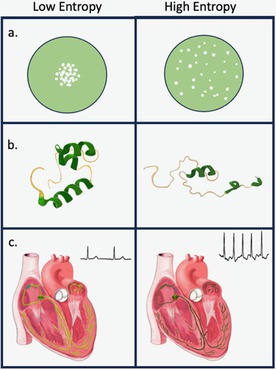

Entropy is a measure of disorder (lack of structure) and randomness (unpredictability) in a system. It is an essential feature of thermodynamics, chemistry, and information theory. The idea of life as an effort to combat entropy was proposed by Erwin Schrodinger in 1944 (Schrödinger 1944). This was extended by Stuart Kauffman into the idea that life emerges from self‐organizing principles in complex dynamic systems (Kauffman 1993). This framework leads to the idea that life exists as a delicate balance between self‐organizing complexity and entropic forces, with aging representing a gradual domination of entropy. Nonetheless, there has been little recognition or research about entropy in human biology and aging.

The 2nd Law of Thermodynamics dictates that in any natural process the total entropy of a system and its surroundings will increase with time. In biological systems, entropy will arise, for example, from random interactions or damage which lead to products that have no biological function or deleterious effects (Gladyshev et al. 2021). This physical disorder will increase with age. Entropy will occur at all levels of biology, for example, disorder of atoms molecules, and molecular structures, like proteins, and tissues such as the cardiac conduction system, manifest as disorder in physiology, as in electrocardiographic (ECG) waveforms (Figure 1).

**FIGURE 1:**
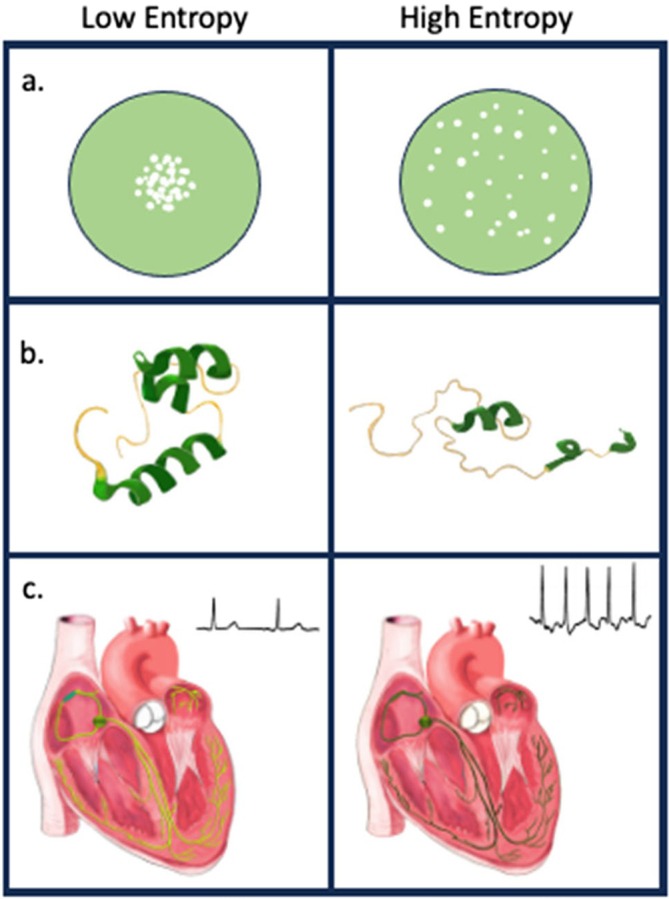
(a) Increasing disorder in the distribution of atoms and molecules. (b) Entropy in proteins promoting loss of normal helical structure. (c) Disorder in the cardiac conduction system within the cardiac muscle leading to altered ECG waveforms. Illustration by Shana Katzman.

Biological systems can partially counter entropy by repairing, metabolizing, replacing or clearing the damage. The rate of aging is a balance of the rate of generation of disorder and damage against the rate of repair and replacement. While processes of repair and replacement may restore order, those restorative mechanisms are themselves subject to the accumulation of disorder and damage. This interaction accelerates the accumulation of disorder and damage (Meyer et al. 2025). The biochemical reactions involved in repair and replacement increase entropy by releasing heat into the surrounding environment.

Entropy has not yet been included as a hallmark of aging; however, it interacts with others. Some hallmarks, such as genomic instability and loss of proteostasis, are at least in part straightforward manifestations of entropy. Others, such as inflammation, stem cell exhaustion, and cellular senescence, may both result from entropy and accelerate it. Stochastic accumulation of oxidative damage in mitochondria with aging might degrade the generation of ATP energy with aging. Epigenetic change is an important hallmark. In mice, inducing stochastic changes in the epigenome caused a loss of information that controls cell structure and function and caused several phenotypic changes of aging (Meyer and Schumacher 2024; Yang et al. 2023) The has been described as the Information Theory of Aging (Lu et al. 2023).

If entropy drives human aging, then several propositions should be true. The amount of entropy will increase with age. Additionally, entropy is a universal property of biological systems, so that entropy in one may be associated with increased risk or severity of aging outcomes in a different system. Testing these predictions and principles in humans requires measurement of entropy in human data, biospecimens, and images. The best known measure of entropy is Shannon's entropy (Figure 2a) (Adami 2004). However, Shannon entropy can only be measured on a distribution (multiple values), a challenge for assigning an entropy value to a single individual, even when there is lots of data about the individual. An alternative is to use Mahalanobis distance (DM), a measure of deviance from population norms that can be measured across many variables with only a single observation per individual (Figure 2b). It can also be conceived of as a manifestation of heterogeneity with aging, measuring an individual's contribution to the population heterogeneity. As entropy increases, deviance from homeostatic norms will follow; the DM approach allows quantification of entropy in multiple data types. Other approaches, such as the recently developed DISCO method, also show promise (Figure 2c) (Hao et al. 2025). DISCO measures the extent to which an individual's biological measurement network deviates from a healthy reference, using distance to the reference covariance structure. DISCO appears applicable to high‐dimensional modalities, including proteomics.

**FIGURE 2:**
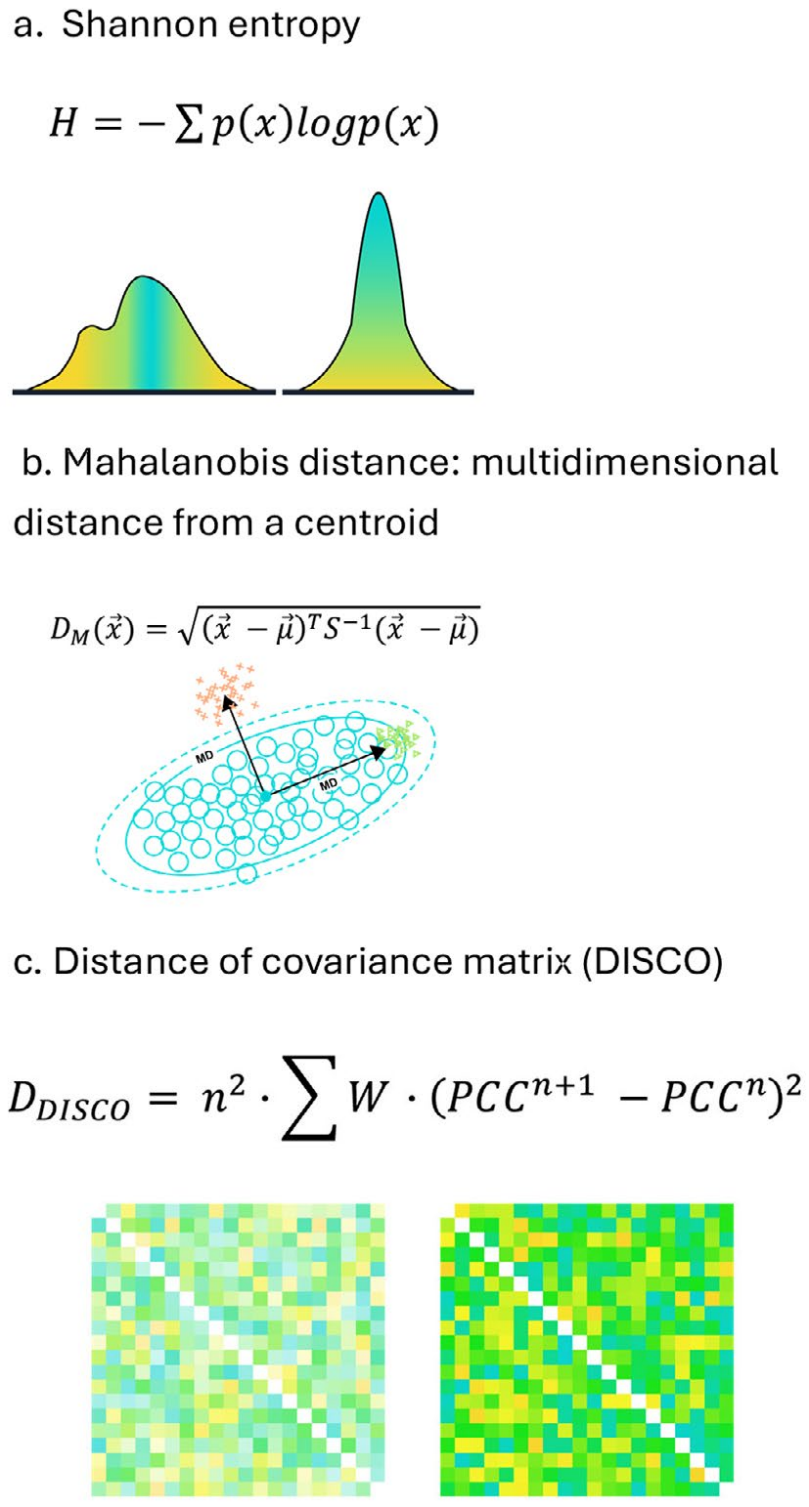
Methods for quantifying entropy. (a) Shannon entropy. (b) Mahalanobis distance: Multidimensional distance from a centroid. (c) Distance of covariance matrix (DISCO).

In this issue of *Aging Cell*, Hong et al. (2025). use DM to measure homeostatic dysregulation as a proxy for entropy, applying it to intervals on ECG tracings in a large community‐based cohort. While this approach has been well established for measuring dysregulation of clinical biomarkers (Li et al. 2015; Dansereau et al. 2019), this is one of the first demonstrations of its use in a different type of data. The approach quantifies how unusual or aberrant an individual's profile of ECG intervals is and is thus expected to be higher as entropy in the system increases.

The study found that the entropy of the ECG intervals increased with age and predicted mortality after adjustment for age and other potential confounders. Surprisingly, ECG entropy also predicted the risk of fractures in that cohort. The association was strong and significant even after adjusting for age and other established risk factors for fracture, including bone density and a history of fractures. The findings of the study satisfy predictions about entropy, expressed as DM: it increases with age. It predicts conditions related to aging: mortality and fractures. Entropy in the cardiac conduction system strongly predicted an aging‐related outcome—fractures—in a very different system. This cross‐system prediction highlights the potential of entropy measures in one system for epidemiological and biological research on aging.

Studies such as Hong et al. (2025) represent the tip of the iceberg, and many crucial questions remain to be answered about entropy and human aging:

- **Can entropy be slowed?** While entropy could be accelerated by various factors (UV radiation, major injuries, etc.), it is less clear how it could be slowed. It has been proposed that decreasing the metabolic rate by caloric restriction, for example, would slow the generation of entropy by reducing random molecular interactions (Demetrius 2004). It has been suggested that exercise may stimulate the clearance of entropic damage by increasing proteostasis, DNA repair, and autophagy (Radak et al. 2005; He et al. 2012). The effects of candidate geroprotector drugs on entropy and damage repair should be investigated as potential mechanisms for slowing aging.
- **Can it be reversed?** Rejuvenation appears to violate the 2nd Law of Thermodynamics by reversing disorder that accumulates with time. Application of transcription factors—Yamanaka factors—can induce the replacement of the epigenome of an old mouse with a young copy of the epigenome with reversal of epigenetic age and several phenotypes (Lu et al. 2023). However, aged cells and tissues accumulate irreversibly disordered and damaged material beyond the epigenome. Thus, rejuvenation also requires that damage be cleared (Sheldrake 2022). Perhaps entire damage bearing cells bearing damage may be degraded and excreted. Alternatively, cells may excrete damage, for example, in exosomes or compartmentalize the damage in a mother with replication of a rejuvenated daughter. Rejuvenation might be consistent with the 2nd Law as these processes involve biochemical reactions that produce energy as heat thereby increasing thermodynamic entropy in the environment.
- **When, if ever, is entropy good**? Can generation of antibody diversity (e.g., via V(D)J recombination (Schatz and Ji 2011)), reactive oxygen species used in signaling (de Magalhaes and Church 2006), or genetic diversity via recombination during meiosis be considered examples of adaptive entropy?
- **How much of what we consider aging is attributable to entropy**? Entropy has the potential to explain much of what we consider aging. How much?
- **What is the causal role of entropy in aging**? The well‐known dynamics of accelerating decline during aging suggest feedback loops and vicious cycles, with entropy undermining resilience and accelerating its own accumulation, but causal roles of specific aspects of entropy have yet to be clarified.
- **What is the relationship between entropy and resilience**? To the extent that entropy is the breakdown in biological information systems, resilience is the capacity to maintain those systems. Does entropy cause loss of resilience? Does resilience prevent entropy accumulation?
- **How integrated is entropy across levels of biological organization**? Are proteomics entropy and ECG entropy correlated, for example? To what extent does entropy propagate across biological scales, and is this equally top‐down and bottom‐up?
- **What is the best way to measure entropy, and how do various measures relate to each other**? Are Shannon entropy, DM, and DISCO correlated? Are there other, better ways to measure entropy at the individual level?

Research on entropy and human aging is in its infancy. Understanding the breadth and importance of entropy and its consequences for human aging will grow as methods for quantifying entropy are applied to many available types of data and images. Analyzing entropy in biological specimens such as gene expression, proteomic data, and tissue samples could determine its importance in the fundamental biology of aging. Such work may establish entropy as a new hallmark of human aging or as a property of several other hallmarks that, together, contribute to human aging.

## Author Contributions

**Steven R. Cummings:** co‐conceived the perspective, wrote most of the 1st draft and several revisions and approved the final version. **Namki Hong:** contributed to the draft, provided figures about methods, approved the final version. **Alan A. Cohen:** Co‐conceived the perspective, revised versions of the paper, approved the final version.

## Conflicts of Interest

The authors declare no conflicts of interest.

## Data Availability

The authors have nothing to report.
